# Mechanisms of immune response and cell death in ischemic stroke and their regulation by natural compounds

**DOI:** 10.3389/fimmu.2023.1287857

**Published:** 2024-01-11

**Authors:** Zhaoyuan Gong, Jing Guo, Bin Liu, Yang Guo, Cuicui Cheng, Yin Jiang, Ning Liang, Mingzhi Hu, Tian Song, Lu Yang, Huizhen Li, Haili Zhang, Xingyu Zong, Qianzi Che, Nannan Shi

**Affiliations:** Institute of Basic Research in Clinical Medicine, China Academy of Chinese Medical Sciences, Beijing, China

**Keywords:** ischemic stroke, inflammatory, immune cells, cell death, natural compounds

## Abstract

Ischemic stroke (IS), which is the third foremost cause of disability and death worldwide, has inflammation and cell death as its main pathological features. IS can lead to neuronal cell death and release factors such as damage-related molecular patterns, stimulating the immune system to release inflammatory mediators, thereby resulting in inflammation and exacerbating brain damage. Currently, there are a limited number of treatment methods for IS, which is a fact necessitating the discovery of new treatment targets. For this review, current research on inflammation and cell death in ischemic stroke was summarized. The complex roles and pathways of the principal immune cells (microglia, astrocyte, neutrophils, T lymphocytes, and monocytes/macrophage) in the immune system after IS in inflammation are discussed. The mechanisms of immune cell interactions and the cytokines involved in these interactions are summarized. Moreover, the cell death mechanisms (pyroptosis, apoptosis, necroptosis, PANoptosis, and ferroptosis) and pathways after IS are explored. Finally, a summary is provided of the mechanism of action of natural pharmacological active ingredients in the treatment of IS. Despite significant recent progress in research on IS, there remain many challenges that need to be overcome.

## 1 Introduction

Stroke is one of the major causes of death and disability worldwide (1). In the past three decades, the global incidence of stroke has increased by 70%, stroke mortality has increased by 43%, disability adjusted life spans lost due to stroke increased by 32%, and the economic burden of all countries has increased. Ischemic stroke (IS) is a severe insufficiency of the blood supply to the brain caused by thrombosis or embolism in the blood supply to the cerebral vessels in the functional area of the brain, resulting in an insufficient oxygen supply to the brain that leads to neuronal death and brain function defects (2). Ischemia induces cell death and neuroinflammation by promoting the production of pro-inflammatory mediators. At present, the number of drugs that can be used to treat IS [such as tissue plasminogen activator (tPA)] is limited, the clinical effect is poor, and the adverse reactions are substantial (3). Therefore, there is an urgent need for further research on IS to find more effective and safe therapeutic agents to prevent or treat IS.

Systemic inflammation, immune responses, and cell death play a key role in the occurrence and development of stroke. Post-ischemic inflammation of the injured brain is characterized by the infiltration of blood immune cells, as well as the interactions between resident microglia and invading blood immune cells (4). After IS, microglia (as resident brain macrophages) and astrocytes are activated in the innate immune system, releasing numerous inflammatory factors (5). Inflammatory factors attract peripheral immune cells to infiltrate the lesion area (6). The mechanisms of action of immune cells are complex, and these cells also interfere with each other, thus forming a complex inflammatory network. This further aggravates systemic inflammation, increases neuronal death and infarct volume, and leads to poor neurological outcomes (7). During ischemia, the blood supply to brain tissue is disrupted, which subsequently promotes a series of pathophysiological reactions leading to different types of cell death, including pyroptosis, apoptosis, necroptosis, ferroptosis, and PANoptosis, the lattermost of which is the crosstalk between pyroptosis, apoptosis, and necroptosis (8). These types of cell death all play roles in the pathogenesis of IS and induce inflammation (9).

This review summarizes—in relation to the immune system, inflammation, and cell death—the mechanisms and pathways involved in IS. In the section on the immune system and inflammation, the pathways and functions of immune cells, including microglia, astrocytes, neutrophils, T lymphocytes, and monocytes/macrophages, in the post-IS inflammatory response are reviewed. In the section on cell death, we review the pathways that mediate pyroptosis, apoptosis, necroptosis, ferroptosis, and PANoptosis. The mechanisms of natural compounds, including salidroside, baicalin, astragaloside IV, and curcumin, in the treatment of IS are also reviewed. Finally, there is a discussion of the potential future directions in this field.

## 2 Role and pathway of immune cells in the inflammatory response to ischemic stroke

**Figure 1:**
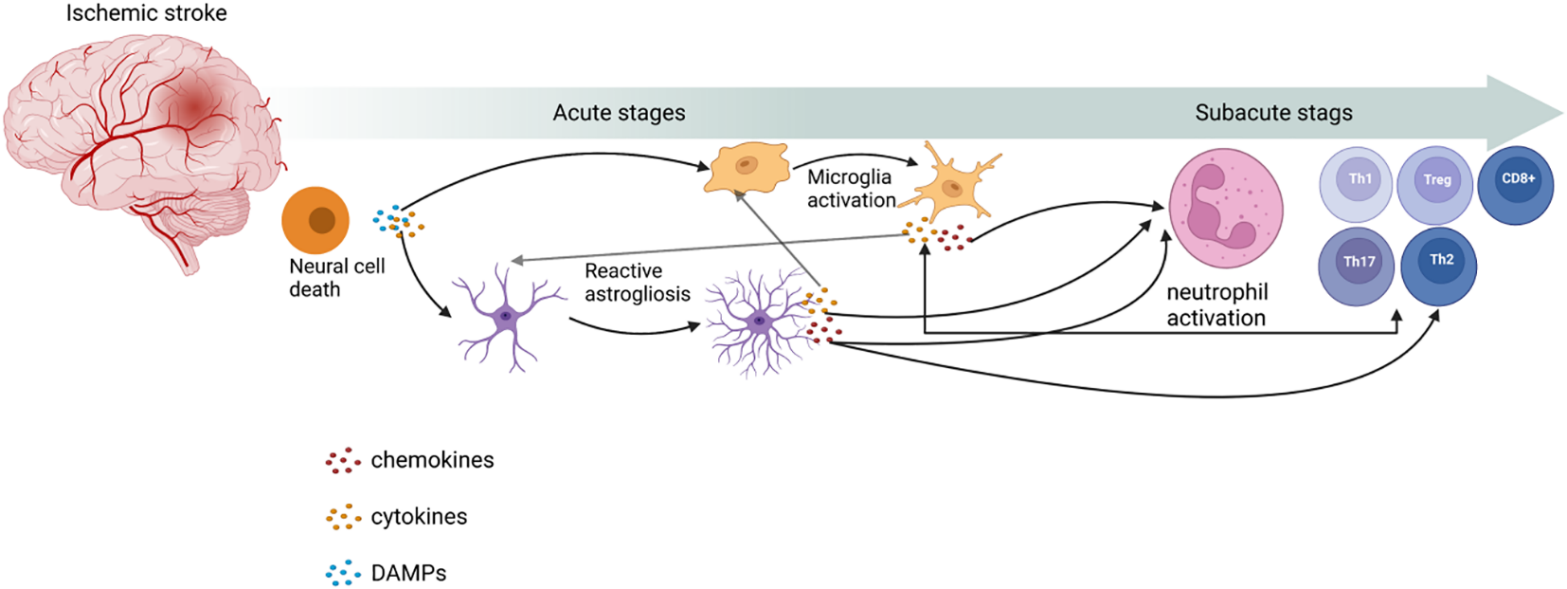
Role of immune cells in ischemic stroke. Post-IS, with the release of damage-associated molecular patterns (DAMPs) and cytokines from neuronal cell death, microglia activation and reactive astrogliosis occur. These processes can release cytokines and chemokines to activate neutrophils and T cells to proliferate and differentiate into different types. This image was created using BioRender.com.

The expression of pro-inflammatory factors marks the beginning of the development of cerebral ischemic inflammation and involves various cell types. The inflammatory response after IS includes two processes, namely early neural injury and late neural repair. Post-stroke inflammation is especially complex, and the interaction of different types of immune cells is crucial as a mediator of neuroinflammation. The immune system can be divided into innate and adaptive immune systems. The innate immune system includes microglia, neutrophils, and astrocytes. Microglia are the first responders in ischemic tissue (10). Moreover, T cells in the adaptive immune system play a role in central nervous system injury and repair (11) (shown in **Table 1**; **Figure 1**).

**Table 1:** Modulators and pathways of immune cell functions.

Immune cells	Type of modulator	Modulator	Model	Regulatory pathway	Effect	Reference
Microglia	Transcription factors	NF-κB	LPS-treated BV2 microglia cells	TLR4/NF-κB	Promote the secretion of pro-inflammatory cytokines	(12)
			LPS-treated BV2 microglia cells	TXA2R/MAPK/NF-κB	Promote the secretion of pro-inflammatory cytokines	(13)
			LPS-treated BV2 microglia cells	MAPK/ERK/NF-κB	Promote the secretion of pro-inflammatory cytokines	(14, 15)
			MCAO mice and OGD/R- BV2 microglia cells	STING/IRF3/NF-κB	Upregulate protein levels of STING, cGAS, p-STING, p-p65, and p-IRF3 in microglia	(16)
			MCAO rats and OGD-treated BV2 microglia cells	Notch/NF-κB	Elevate Notch-1 and Delta-1 expression in microglia; increase mRNA expression of TNF-α, IL-1β and iNOS	(17, 18)
	Transcription factors	STAT family members	Hypoxia-BV2 microglia cells, MCAO/R rats, and OGD/R microglia	JAK/STAT pathway	Elevate the expression of NF-κB	(19, 20)
	Ion channel protein	HV1	Mice lacking Hv1 (Hv1-/-)	HV1/NOX/ROS	Elevate the expression of ROS	(21)
	Ion channel protein	Kv1.3	MCAO/R rats, OGD/R primary microglia, ICV-LPS mice, microglia (adult brains)	/	Elevate the expression of pro-inflammatory cytokines, activate NLRP3 inflammasome	(22, 23)
	Gene	H19	MCAO mice and OGD/R- BV2 microglia cells	H19 siRNA/HDAC1	Elevate the expression of pro-inflammatory cytokines	(24)
	Gene	miRNA-155	LPS-treated BV2 microglia cells	miR-155/SOCS1	Elevate the expression of pro-inflammatory cytokines,	(25)
	Cytokine	IL-4	Microglia/macrophage polarization BV2 cells	IL-4/JAK1/STAT6	Alleviate neuroinflammation	(26)
	Transcription factors	PPARγ	pMCAO rats	IL-4R/STAT6/PPARγ	Improve neurological function	(27)
	Transcription factors	Nrf2	tFCI rat	AMPK/Nrf2	Anti-inflammatory	(28)
			CUMS mice, LPS/ATP-treated BV2 cells	Nrf2/HO-1/NLRP3	Upregulate the expression of Nrf2, HO-1, downregulate the expression of NLRP3	(29)
	Transcription factors	STAT family members	tMCAO/R mice	STAT1/STAT6	Lead to neuronal survival, and neurological functional recovery	(30)
	Gene	miRNA-124	TBI rat	miRNA-124/TLR4	Inhibit TLR4	(31)
			MCAO mice	miRNA-124/STAT3	Inhibit astrocyte proliferation, decrease Notch 1 expression and increase Sox2 expression in astrocytes	(32)
	Gene	FAM19A3	MCAO mice	/	Attenuate cerebral ischemia	(33)
Astrogliosis	Receptor	P2Y_1_R	tMCAO rats	P2Y_1_R/NF-κB	Promote the secretion of pro-inflammatory mediators, activates the NF-κB pathway,	(34)
			TBI mice	TNF-α, IL-1β, IL-6 (Microglia)/P2Y_1_R	P2Y_1_R downregulation, GFAP and p-STAT3 upregulation	(35)
	Receptor	TLR-4	LPS-treated rodent brain astrocyte cultures	TLR-4/MyD88/NF-κB TLR-4/MAPK/JAK1/STAT1	Promote the secretion of pro-inflammatory cytokines, chemokines	(36)
	Protein	p38 MAPK	MCAO mice and primary astrocyte cultures	MAPK	Increase GFAP expression	(37)
	Protein	Notch	MCAO mice	Notch1/RBP-J	Increase GFAP expression and promote reactive astrogliosis	(38)
	Transcription factors	STAT family members	MCAO mice and OGD-treated primary astrocytes	DRD2/CRYAB/STAT3	Promote the secretion of pro-inflammatory cytokines and astrocytic activation	(39)
	Protein	Complement system	Rat primary astrocytes	C3a/C3aR, C5a/C5aR	Decreases the production of cAMP and increase in intracellular calcium concentration	(40)
Neutrophils	Chemokine	CCL3	tMCAO mice	CCL3/CCR1 and CCR5	Recruit neutrophils	(41)
		CXCR1/2	MCAO/R rats	CXCL8/CXCR1/2	Activate neutrophil, recruit neutrophil	(42)
		CCR5	MCAO rats	CKLF1/CCR5	Mediate neutrophils infiltration, migrate neutrophils may via Akt/GSK-3β pathway	(43)
	Transcription factors	PPARγ	MCAO mice	RXR/PPARγ	Increase brain infiltration of N2 neutrophils	(44)
	Receptor	TLR4	t-PA induced HT rats	HMGB1/TLR4/NF-κB	Mediate neutrophil infiltration, disrupt BBB integrity	(45)
			Human neutrophils; FeCl3-induced CAT rats	TLR4/MyD88/MAPKs	Promote thrombogenesis, induce NETs formation	(46)
T cell CD8^+^	Protein	FasL	MCAO mice	FasL/PDPK1	Promote cytotoxicity, apoptosis of neurons, and ischemic neurological dysfunction	(47)
Treg	Transcription factor	Foxp3	Rats, LPS-treated microglia/macrophages	IL-2/Foxp3/STAT5	Sustain Foxp3 expression and Treg-cell identity	(48)
	Chemokine	CXCL14	MCA and CCA rats, primary cortical	HIF-1α/CXCL14	Induce Treg differentiation, promote Treg accumulation, reduce infarct volume	(49)
	Cytokine	IL-2	Five different types of Treg cells from human umbilical cord blood	IL-2/JAK/STAT	Enhance IL-10 production and IL-10 mRNA expression	(50)
CD4^+^ Th1	Transcription factor	T-bet	Gene knockout mice and wild-type mice	IL-2,IL-12/IL-12Rβ2/T-bet	Promote TH1 differentiation	(51)
Th2	Transcription factor	GATA3	CD4^+^ splenic T cell from mice	IL-2/STAT5/GATA3	Drive Th2 differentiation, induce and maintain IL-4Rα,	(52)
Th17	Transcription factor	RORγt	Gene knockout mice and wild-type mice	IL-6/STAT3/RORγt	Direct Th17 differentiation, induce IL-17 and IL-17F expression	(53)
M1 macrophages	Receptor	P2X4R	MS-treated P2X4R knockout mice and wild-type mice	/	Increase IL-1β, IL-6, TNF-α mRNA levels,	(54)
M2a macrophages		IL-13	pMCAO mice and RAW 264.7 macrophages		Enhance the expression of M2a alternative activation markers (Arg1 and Ym1), increase IL-6 and IL-10 levels, decrease neuronal cell death	(55)

nuclear factor NF-kappa-B (NF-κB), thromboxane A2 (TXA2R), Janus kinase (JAK), phosphorylated-JAK (p-JAK), lipopolysaccharide (LPS), voltage-gated proton channel (Hv1), nicotinamide adenine dinucleotide phosphate oxidase (NOX), reactive oxygen species (ROS), voltage-gated potassium channel (Kv1.3), NACHT, LRR and PYD domain-containing protein 3 (NLRP3), histone deacetylase 1 (HDAC1), suppressor of cytokine signaling 1 (SOCS1), nuclear factor erythroid 2-related factor 2 (Nrf2), AMP-activated protein kinase (AMPK), peroxisome proliferator-activated receptor γ (PPARγ), interleukin-4 (IL-4), signal transducer and activator of transcription (STAT), sphingosine 1-phosphate receptor (S1PR), sphingosine 1-phosphate (S1P), transient focal cerebral ischemia (tFCI), transient middle cerebral artery occlusion-reperfusion (tMCAO/R), purinergic receptor type 1 (P2Y_1_R), phosphorylated-nuclear factor NF-kappa-B p65 subunit (p-RelA), recombining binding protein suppressor of hairless (RBP-J), dopamine D2 receptor (DRD2), αB-crystallin (CRYAB), adenosine 3’,5’-cyclic monophosphate (cAMP), glutamic acid-lysine-arginine (ELR), chemokine-like factor 1 (CKLF1), CC chemokine receptor 5 (CCR5), hemorrhagic transformation (HT), carotid artery thrombosis (CAT), 3-phosphoinositide-dependent protein kinase-1 (PDPK1), tumor necrosis factor ligand superfamily member 6 (FasL), right middle cerebral artery (MCA), bilateral common carotid artery (CCA), interleukin 12 receptor β2-chain (IL-12Rβ2), chromodomain helicase DNA-binding protein 4 (Chd4), IL-4 receptor alpha-chain (IL-4Rα), GATA-binding factor 3 (GATA3), interleukin-17F (IL-17F). /, not applicable.

### 2.1 Brain immune cells

#### 2.1.1 First line of defense: microglia

Microglia, as resident CNS macrophages, play a dual role of neurotoxicity and protection in IS. When brain injury occurs, numerous DAMPs and cytokines are released, and microglia are activated with the death of nerve cells in the central area of the infarct (5); this, together with the activation of macrophages from blood-derived monocytes, constitute the innate immune response, which is the first line of defense (56). During systemic inflammation, microglia are involved in tissue damage and repair, respectively (57).

Previously, the pro-inflammatory microglia phenotype was termed M1, while the anti-inflammatory phenotype was termed M2. Since then, microglia have been demonstrated to exist in a wide range of activated states. For example, several *in vivo* studies failed to even find pure M1 or M2 states. In the same cell and at the same point in time, these “microglial markers” have multiple overlapping phenotypes. Therefore, a binary M1/M2 characterization is not sufficient for defining the inflammatory characteristics of microglia (58), and a systematic and careful nomenclature will greatly benefit the biological study of microglia. The current view is that moderate and precise terms should be used to properly understand the state of microglia. Using markers (genes or proteins) to identify populations of cells may be a solution, but they cannot be used as a readout of cell function (59). Wahane et al. combined transcriptomics and single-cell RNA sequencing to reveal a wide range of microglial states after spinal cord injury. The transcription profiles were diverse, with each transcription profile comprising four transcription subtypes. Furthermore, RNA-seq showed a well-defined temporal trajectory of IAM (injury-activated microglia and macrophages) gene programs over several days, including 3 days proliferation and motility, 7 days axon chemical attraction and ion channel activity, and 14 days extracellular matrix (ECM) recombination. The data also found that (i) phagocyte genes were still induced at 14 days to support the durable repair function of IAM; (ii) there was a high expression of ECM genes and nutrient factors; and (iii) the anti-inflammatory gene set signature was enriched at all stages. These three phenomena indicate that IAM has a long-lasting repair function. RNA-seq did not show significant changes in M1/M2 genes in bone marrow cells, and scRNA-seq further revealed their heterogeneous expression patterns in bone marrow subclusters. This further demonstrates the limitations of the conceptual dichotomy of pro-inflammatory and anti-inflammatory phenotypes (60).

The involvement of microglia in tissue damage reaches its peak 3–5 days after ischemic injury. At this stage, microglia play a harmful role, mainly by destroying the blood brain barrier (BBB), aggravating brain edema, and promoting neuronal apoptosis by producing and secreting many inflammatory mediators (61). The release of pro-inflammatory cytokines leads to secondary brain injury, while microglia have been shown to exhibit repair functions for nearly 14 days after injury (62). Microglia play a primarily protective role that promotes the regression of inflammation by secreting IL-4, IL-10, and transforming growth factor (TGF)-β, thereby indirectly preventing inflammation-induced damage to the blood brain barrier (BBB). The regulatory pathways of microglial cell polarization can be divided into four categories: 1) transcription factors; 2) receptors; 3) ion channels; and 4) gene modulators (shown in **Table 1**). Moreover, microglial phagocytosis is a double-edged sword in immune inflammation and stroke recovery. Microglia have been shown to exhibit phagocytosis, and their ability to clean neuronal debris reduces brain damage after stroke (5). Microglia invade the ischemic site of stroke earlier than macrophages, and they are the main phagocytes for the first three days after stroke (63). After ischemia, microglia infiltrate the injured brain tissue, engulfing living and dead neurons, myelin debris, apoptotic cell debris, endothelial cells, and leukocytes. During pathological cases, microglial phagocytosis can be initiated by specific “eat me” signals on specific cell types and their corresponding receptors (64).

As shown in **Table 1**, many pathways and mediators have been confirmed to regulate the activation of microglia. For example, NF-κB and IL-4 as regulators promote activation of microglia, respectively, and are the most extensively studied and clearly researched mediators. Some conflicting results have been found in studies on the regulatory effects of TLR and STAT, and further research is necessary to clarify their roles (65). Most of these signaling pathways overlap to varying degrees and do not appear to work independently, but rather synergistically, resulting in an inflammatory maelstrom (66). Many studies have shown that targeting microglia can effectively treat IS (67). Further research on microglial activation signal pathways after IS will help identify effective drugs that inhibit microglial activation and prevent neuroinflammation mediated by microglial activity. Therefore, in future studies, it is crucial to identify appropriate targeted intervention drugs according to the role of microglia at different times.

#### 2.1.2 Dual regulatory effects of astrocytes

Astrocytes, the most abundant neuroglial cells in the brain, are essential housekeeping cells that maintain the central nervous system. Astrocytes play, as do microglia, a dual role in the pathophysiology of IS (68). After IS, damaged cells produce and release cytokines and DAMPs to stimulate receptors of astrocytes and change their phenotype. A few minutes after IS, due to reactive astrocyte proliferation, astrocytes respond to various inflammatory factors (including TGF-α, ciliary neurotrophic factor, IL-1, IL-6, and kallikrein-related peptidase 6) released by ischemic/hypoxic cells, and are subsequently activated and reproduced (69, 70). Reactive astrogliosis occurs in the peri-infarct region, and a glial scar is formed to maintain CNS homeostasis and wall off the lesion (71). During this process, astrocytes display cellular hypertrophy, proliferation, and increased expression of intermediate proteins, including glial fibrillary acidic protein (GFAP), vimentin, and nestin (72). After experiencing reactive astrogliosis, astrocytes produce and release pro-inflammatory cytokines (such as IL-6, TNF-α, IL-1α, IL-2β, and IFN-γ), chemokines (such as CXCL1/10 and CCL2/3/5), important sources of ATP, and free radicals such as NO, superoxide, and peroxynitrites (73). Thus, the activation of microglia and infiltration of white blood cells are enhanced (74). Studies have discussed the two subtypes of reactive astrocytes as A1 and A2. The A1 subtype includes astrocytes induced by IL-1α, TNF-α, and complement component subunit 1q (C1q) secreted by activated microglia. A1 subtype astrocytes induce neuronal and oligodendrocyte death. The A2 subtype can secrete IL-2, IL-10, and TGF-β, thus accelerating the regression of inflammation (75). Additionally, the A2 subtype can play an inflammatory and neuroprotective role by secreting neurotrophic factors, neuropoietic cytokines, and growth factors (76).

The response of astrocytes to Injury is a major determinant of the outcome after stroke. The gene expression of A2 astrocytes dominates the expression of A1 astrocytes (77). The JAK/STAT3 signaling pathway was found to be an important switch controlling many molecular and functional changes in reactive astrocytes *in vivo* and *in vitro* (78). However, the roles of all these molecules and pathways need to be further validated in future research (79, 80). In addition, the complexity of the multiple roles of complement protein and receptor expression in astrocytes has only recently been studied. Further *in vivo* and *in vitro* research is needed to determine astrocyte pathways and effects to determine targeted treatment strategies.

### 2.2 Peripheral immune cells

#### 2.2.1 Recruitment and infiltration of neutrophils

Increased numbers of leukocytes have been found to be a marker of the inflammatory response in IS. Among various types of leukocytes, neutrophils are the first to respond to ischemic brain injury (81). An *in vivo* study found that neutrophils were found in leptomeninges and cerebral parenchyma 6 hours and 12 hours, respectively, after permanent middle cerebral artery occlusion (pMCAO) (82). Their recruitment reached a peak on days 1-3 and gradually decreased over time (83). Neutrophils produce extensive weblike structures of DNA (neutrophil extracellular traps, NETs) that reached their peak 3-5 days after the transient middle cerebral artery occlusion (tMCAO) (84). These NETs have been associated with inflammation (85). After cerebral ischemia, neutrophils undergo conformational changes due to the presence of many adhesion molecules, which helps them to migrate through blood vessel walls to the brain tissue. Activated microglia and astrocytes release chemokines (such as CXC and CC) to promote neutrophil activation. These chemoattractants bind to the C-C chemokine receptor 5 (CCR5) and C-X-C chemokine receptor 1 (CXCR1) on the surface of neutrophils, making neutrophils the first blood-derived immune cells to migrate to damaged brain tissue (86). Neutrophils are attracted to the ischemic region by chemokines, and then infiltrate damaged brain tissue soon after injury, which aggravates inflammation (87).

Traditionally, neutrophils have been considered the main mediators of harmful inflammatory responses in IS (88). However, a significant amount of evidence suggests that neutrophils can obtain different phenotypes. As in the case of microglia, it is believed that some neutrophil subsets show different characteristics. The response phenotype of neutrophils to the ischemic environment, and the interaction between neutrophils and endothelial adhesion molecules has shifted from protective N2 to the injurious N1 phenotype (89). *In vivo* and *in vitro* studies have shown that PPARγ and TLR4 mediate the N2 phenotype of neutrophils (90, 91). However, research on the functional changes and biomarkers of the N1/N2 phenotypes of neutrophils after IS are not sufficient. This also leads to a shortage of known pathways. Therefore, further research is needed on the role of neutrophils in the inflammatory response after IS.

#### 2.2.2 Conflicting roles of T lymphocytes

T lymphocytes play an important role in the process of nerve damage and repair in the late stage of IS. In the acute phase of IS, T cells chiefly react in an antigen-independent manner and are closely related to the development of the infarct volume. After 3-7 days, the T cell response gradually transforms into antigen-dependent antigen recognition (92). Brain-derived antigens are recognized by T cell receptors (TCRs) on the surface of naïve T cells. Then, T cells migrate to the brain parenchyma through cell adhesion molecules (such as P-selectin, E-selectin, VCAM-1, and ICAM-1) and chemokines. Ultimately, adaptive immune responses exacerbate ischemia-reperfusion (I/R) injury. *In vivo* studies involving ischemic rats demonstrated that by day 3 after ischemia, many T cells infiltrated the peripheral areas around the lesion and surrounded the infarct center, and the number of T cells increased between days 3 and 7 (93). According to different functions, T cells have multiple types marked by CD3 expression, including CD8^+^ cytotoxic T lymphocytes (CTL), CD4^+^ T helper (Th) cells, regulatory T cells (Treg), and gamma delta (γδ) T cells (94). The different roles of different types of T cells are already known, but the specific mechanism of T cell function after IS still needs further research.

CD^4+^ T cells, as the main effector T cells, regulate brain inflammation by producing cytokines (95). The signals derived from T cell and co-stimulatory T cell receptors and extracellular cytokines determine the phenotype of Th cells. Cytokine signals are received through multimeric receptors and propagated largely through Janus kinase/signal transducer activator of transcription (JAK/STAT) signaling pathways (96). Th cells can be divided into Th1 and Th17 (pro-inflammatory), and Th2 and Treg (anti-inflammatory) based on their cytokine secretion profile. Th1 and Th17 cells produce IL-1, IFN-γ, IL-17, IL-22, and other cytokines. Th2 and Treg cells produce IL-4, IL-10, and TGF-β (97). Different types of CD4^+^ T cells have their own specific transcription factors that play a crucial role in their differentiation, maintenance, and function (98).

CD8^+^ T cells can play a cytotoxic role through antigen recognition of the TCR and subsequent release of granzyme and perforin, forming pores on target cells and inducing apoptosis (99). Selvaraj et al. investigated the role of CD8^+^ T cells in stroke by establishing a tMCAO mouse model. The results showed that CD8^+^ T cells had an adverse effect in the chronic phase after stroke. At 30 days, there was an increase in the number of ipsilesional CD8^+^ T cells, revealing its association with deterioration in mouse functional outcomes (100). In recent years, there have been studies on CD^8+^ T cells inducing neuronal apoptosis through the FasL/PDPK1 pathway, but their mechanism of action after IS remains unclear.

γδ T cells do not require antigen recognition to activate and are detected in infarcts 6 hours after ischemia. During the onset of IS, γδ T cells mainly secrete cytokines such as IL-17, IL-21, IL-22, and IFN-γ through receptors to protect the barrier from infection and exacerbate inflammation (101). Arunachalam et al. found that Vγ6^+^/CCR6^+^ γδ T cell subtypes are the main source of IL-17 (102). However, few studies have paid attention to the signaling pathways present in γδ T cells after activation by ligands that bind to their receptors. The low number of γδ T cells, difficulty in extraction, and lack of cell lines may be the reasons for this lack of research.

#### 2.2.3 Double-edged sword: monocytes/macrophage

The role of monocytes and macrophages in ischemic stroke is the same as that of microglia. Post-IS, pro-inflammatory monocytes infiltrate the inflamed brain, where they differentiate into macrophages that are morphologically indistinguishable from the local microglia (103). In contrast to the rapid microglial response, macrophages are rarely detected within the first 48 hours. Their level gradually increases, with a peak during the first week after stroke (104). On day 3 after stroke, the phenotypes of monocytes were found to change from the predominantly pro-inflammatory M1 to the anti-inflammatory M2 phenotype, indicating a functional shift from an enhanced immune response to inflammation resolution (105). Transcriptomic analysis of macrophages has shown that infiltrated macrophages on day 5 after stroke promote an effervescent increase and inflammation resolution after ischemic stroke (106). M2 macrophages can be further subdivided. For example, studies have classified macrophages as M2a, M2b, and M2c (107), while other authors have also classified them into an M2d subtype (108). All four M2 macrophage subtypes acquired enhanced phagocytosis and expressed IL-10, contributing to the resolution of inflammation (61). In a mouse model of ischemic stroke, researchers found that inflammatory activity peaked at 72 hours. Microglia produce relatively high levels of reactive oxygen species and TNF, while monocytes are major IL-1β producers. Although microglia show enhanced phagocytosis activity after stroke, monocytes have a significantly higher phagocytosis capacity at 72 hours (104).

Most M2 macrophages derived from monocytes can protect the blood-brain barrier from ischemic damage through vascular remodeling, physical attachment, and regression of inflammation (109). M2a macrophages express various anti-inflammatory and neurotrophic factors, such as arginase 1 (Arg1) and insulin-like growth factor-1. M2c macrophages increase the expression of TGF-β, CD163, and sphingosine kinase. However, M2b macrophages increase the production of pro-inflammatory factors, including IL-1β, IL-6, and TNF-a, which may enhance inflammation and increase blood-brain barrier permeability early in IS (110). M2d macrophages secrete VEGF-A and TNF-α, all of which are detrimental to the blood-brain barrier integrity in IS (111).

### 2.3 Mutual coordination between immune cells in ischemic stroke

#### 2.3.1 Crosstalk between microglia and astrocytes

The interaction between activated microglia and astrocyte has a critical role in the process of neuroinflammation (shown in **Table 2**). In the first 6 hours after cerebral ischemia, microglia are first activated by pathogens or injury through TLR4, and release inflammatory mediators (112, 113). At the same time, astrocytes dependently activate TLR2, TLR3, and TLR4 to respond (114). The “molecular signal” (IL-1, TNF-α, and C1q) released by microglia can convert astrocytes into a neurotoxic A1 phenotype. For example, Dr. Ben Barres’ lab, using single-, dual-, and triple-gene knockout mice, pioneered the discovery that activated microglia secreting IL-1α, TNF-α, and C1q together are necessary and sufficient to induce A1 astrocytes (75). These neuroinflammatory reactive astrocytes lose many of their stereotypical physiological functions and secrete one or more unknown factors with strong toxicity to neurons and oligodendrocytes (115). Tarassishin et al. showed that human astrocytes and reactive astrogliosis are highly sensitive to IL-1β but unresponsive to lipopolysaccharide (LPS) stimulation. In human astrocytes, IL-1 induced both A1 and A2 responses (116). Glucagon-like peptide-1 receptor (GLP1R) is highly expressed in microglia, and is also expressed in astrocytes and neurons at reduced levels. Some studies have found that GLP1R agonists can directly prevent microglia-mediated astrocyte transformation into the A1 neurotoxic phenotype and have neuroprotective effects (117). The interleukin-1 family member interleukin-33 (IL-33) is produced by developing astrocytes, and it mainly signals to microglia and promotes synaptic phagocytosis of microglia under physiological conditions. IL-33 also drives microglia-dependent synaptic depletion *in vivo*. The transcriptomes of acutely isolated microglia from IL-33^−/−^ animals showed 483 significantly altered transcripts, including reduced expression of NF-κB targets (e.g., Tnf, Nfkbia, Nfkbiz, and Tnfaip3) (118). IL-15 is also the mediator of crosstalk between astrocytes and microglia, thus aggravating brain damage after intracerebral hemorrhage. Shi et al. established a transgenic mouse model targeting IL-15 expression in astrocytes and found that the accumulation of microglia near astrocytes in the tissue around the hematoma increased after brain injury. The expression of biomarkers in M1 microglial cells increased significantly (119).

**Table 2:** Cytokines in mutual coordination between immune cells.

Cytokines	Main producer	Effect immune cell	Role in ischemic stroke
TNF-α	M1 microglia, Th1 cell	T cell, microglia, and astrocyte	Activate astrocytes, accelerate the polarization of Th1 cells, mediate endothelial necrosis, promote the destruction of BBB, promote M1 polarization
IL-1β	Monocytes/macrophages M1 microglia	Astrocyte	Activate astrocytes, aggravate the dysfunction of BBB, stimulate the activation of microglia, and promote the apoptosis of damaged cells
IL-6	M1 microglia, astrocytes,	T cell, microglia, astrocytes,	Recruit and induce differentiation of Th17 cells, promote proliferation and activation of microglia and astrocytes, aggravate the damage effect
IL-12	M1 microglia	T cell	Accelerate the polarization of Th1 cells
IL-15	Astrocytes	T cell, microglia,	Increase the number of CD8^+^T cells and activated brain infiltrating CD4^+^T cells, promote the differentiation and accumulation of M1 microglia, and aggravate ischemic brain damage,
IL-17	γδ T cells, astrocyte, and Th17 cells	Neutrophil	Promote neutrophil recruitment to the ischemic hemisphere, upregulate neutrophil-mobilizing cytokines and chemokines,
Anti-inflammatory			
IL-4	CD4^+^, Treg, Th2 cell	Astrocyte, microglia	Promotes M2 polarization of microglia, inhibits pro-inflammatory cytokines (IL-1β, TNF-α)
IL-10	M2 Microglia Th1, Th2, Treg and astrocyte	Microglia, T cell,	Mediate the function of Th2 cells to reduce infarction lesions, inhibit cell apoptosis, and drive M2 Microglia polarization,
IL-33	Astrocytes, Th2 cell	Treg cell, microglia	Expand Treg cell and induce IL-4 secretion, activate M2 microglial polarization, and reduce astrocytic activation

References are shown in the text.

#### 2.3.2 Crosstalk between glial cells and peripheral immune cells

Astrocytes are the bridge between infiltrating T lymphocytes and neurons during cerebral ischemia. *In vivo* knockdown of interleukin-15 (IL-15) in astrocytes alleviates ischemic brain damage. Decreased levels of CD8^+^ T cells were also found in mice with knockdown of the IL-15 receptor α or blockade of cell-to-cell contact. Subsequent studies further confirmed the role of IL-15 from astrocytes on T cells. At the same time, a lower number of activated brain infiltrating CD4^+^ T cells were also found in Il15^−/−^ mice (120). Astrocytes, γδ T cells, and Th17 cells are the main sources of interleukin-17 (IL-17) after IS. The main function of IL-17 involves coordinating local tissue inflammation by upregulating pro-inflammatory and neutrophil-mobilizing cytokines and chemokines. Kang et al. established a mouse model with specific deletions of key components of IL-17 signaling in various immune cells. It was found that astrocytes were crucial in IL-17-mediated white blood cell recruitment (121). Astrocyte-derived CXCL-1 acts as a key mediator of IL-17-initiated neutrophil chemotaxis in stroke. IL-17 secreted by γδ T cells has also been reported to attract neutrophils to the site of injury (122). Subsequently, reactive microglia engulf neutrophils in the periphery of ischemic lesions, while the local microglia loss and dystrophy occurring in the ischemic core are associated with the accumulation of neutrophils, first in perivascular spaces and later in the parenchyma (123). Following IS, central nervous system injury can trigger the release of IL-33 from astrocytes. Ito et al. found that many Treg cells accumulated in the brain of mice dependent on IL-33 after IS. The chemokines CCL1 and CCL20 drive penetration into the brain. This helps with neurological recovery in the chronic phase of ischemic brain injury (124). In the MCAO mouse model, IL-33 treatment increased the number of Treg cells in the ischemic brain. IL-33 was shown to increase the levels of anti-inflammatory cytokines in serum and brain tissue (125). IL-33 also enhanced M2 polarization marker expression in microglia. Activation of the IL-33/ST2 axis led to polarization of M2 microglia, which provided protection for ischemic neurons in an IL-10 dependent manner (126).

The crosstalk between M1 microglia and Th1/Th17 cells plays a pro-inflammatory role and contributes to brain injury. The crosstalk between M2 microglia and Th2/Treg cells plays an anti-inflammatory role and helps with brain recovery. M1 microglia secrete IL-12 and TNF-α, which induce Th1 cells, and these two types of cells work together to promote inflammation. The M1 polarization promoted by Th1 cytokines (TNF-α and IFN-γ) is associated with classic activation (127). M1 microglia secrete IL-6 and IL-23, which recruit Th17 cells and induce their differentiation (128). Th2 cells secrete IL-4 and IL-10, while Tregs secrete IL-10, further driving M2 polarization, inhibiting inflammation, and promoting tissue repair. IL-33 is suppressed in human stroke, resulting in an insufficient Th2-type response driven. In human T cells, IL-33 treatment induced IL-4 secretion while reducing astrocyte activation and increasing the number of M2 microglia (129).

## 3 Cell death in ischemic stroke

**Figure 2:**
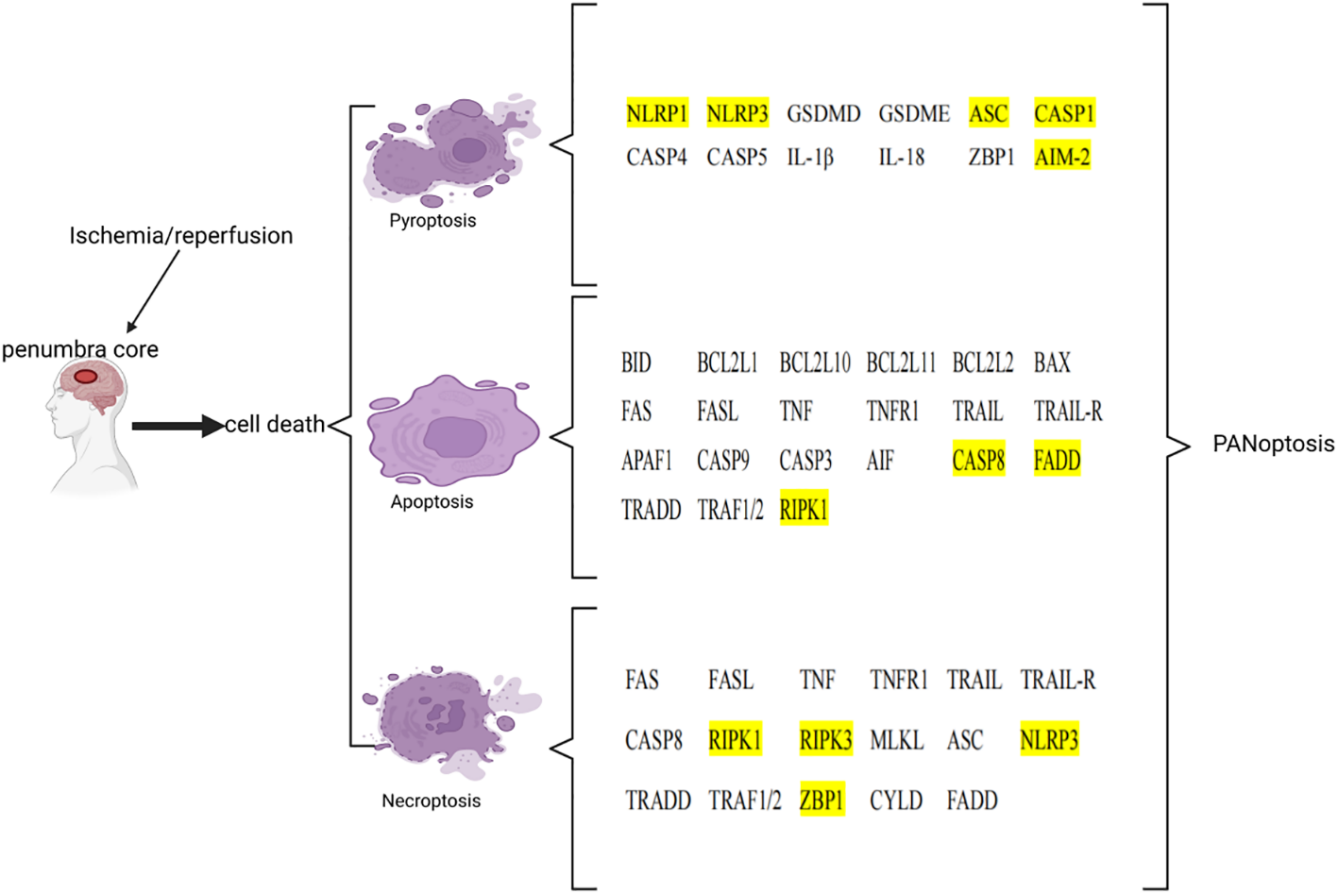
List of key genes involved in pyroptosis, apoptosis, and necroptosis in ischemic stroke. The genes involved in PANoptosis are marked in yellow. This image was created using BioRender.com.

After ischemia, hypoperfusion of brain tissue leads to a decrease in oxygen, ATP, and glucose, which leads to cell death over time. Ischemic tissue can be functionally divided into irreversibly injured infarcted core tissue and peripheral ischemic penumbra tissue. The infarct core is composed of dead or dying tissues and is located in the central area of the infarct area. In the penumbra, this depletion hampers cellular physiological functioning but does not induce an irreversible change. Neuronal death in IS involves a variety of cell death pathways. Apoptosis, pyroptosis, necroptosis, and PANoptosis are four key cell death pathways (**Table 3**; **Figure 2**).

**Table 3:** Cell death and pathways.

Cell death	Model	Type of modulator	Modulator	Pathway	Reference
Pyroptosis	MCAO/R mice, primary microglial	PRR	NLRP3	NF-κB/NLRP3	(130)
	MCAO/R rats, OGD/R-treated SH-SY5Y cells	PRR	NLRP3	NLRP3/Caspase-1/GSDMD	(131)
	MCAO rats, OGD/R-treated neurocytes	PRR	AIM2	lncRNA MEG3/miR-485/AIM2	(132)
	MCAO/R rats,	Protein	GSDMD	GSDMD/caspase-1	(133)
	MCAO rats	PRR	NLRP1	miR-9a-5p/NLRP1	(134)
	ICH mice	Adaptor protein	ASC	Asc/GSDMD/Caspase-1	(135)
Apoptosis	MCAO mice, OGD/R-treated primary cultured mouse embryonic cortical neurons	Pro-apoptotic protein	P53	p53/Bcl-2/Bax	(136)
	CIR rats	Pro-apoptotic protein	P53	p53/Bax/Cytochrome C/Caspase-3	(137)
	MCAO/R rats	Pro-apoptotic protein	ERK	ERK/JNK/p38/Bim	(138)
	MCAO mice, OGD/R-treated PC12 cells	Pro-apoptotic protein	ERK	ERK1/2/CREB/BCL-2	(139)
	FI/R mice	Pro-apoptotic protein	JNK	JNK/Bim, Bax	(140)
	MCAO/R mice, SH-SY5Y cell	Pro-apoptotic protein	NF-κB	NF-κB/Bim/caspase-3	(141)
	MCAO rats	Pro-apoptotic protein	Notch/HIF-1α	Notch/HIF-1α/Bcl-2/Bax	(142)
	OGD-treated cortical cultures, TNFR1 knock-out mice	Receptor	TNFR1	TNFR1/TNF-α	(143)
Necroptosis	MCAO rats	Kinase	RIPK1/RIPK3	RIPK1/RIPK3/MLKL	(144)
	I/R rats and H/R-treated H9c2 rat cardiomyoblast cells	Kinase	RIPK1/RIPK3	TNF-α/RIP1/RIP3/MLKL	(145)

middle cerebral artery occlusion (MCAO), oxygen-glucose deprivation/reperfusion (OGD/R), pattern recognition receptors (PRR), NOD-like receptors containing pyrin domains (NLRPs), absent in melanoma 2 (AIM2), long non-coding RNA (lncRNA) maternally expressed gene 3 (MEG3), gasdermin D (GSDMD), microRNA-9a-5p (miR-9a-5p), intracerebral hemorrhage (ICH), Ag phosphatidylinositol 3-kinase (PI3K), cerebral ischemia-reperfusion (CIR), extracellular signal-regulated kinase (ERK), cyclic AMP-responsive element-binding protein (CREB), focal ischemia and reperfusion (FI/R), genetically deficient mouse embryo fibroblasts (MEFs), mesenchymal stem cells (MSCs), ischemia/reperfusion (I/R), hypoxia/reoxygenation (H/R).

### 3.1 Pyroptosis, apoptosis, necroptosis, and PANoptosis

#### 3.1.1 Pyroptosis

Pyroptosis is a form of regulatory necrosis mediated by caspase-1 and is mainly seen in the ischemic penumbra. Pyroptosis can be divided into inflammatory and non-inflammatory pathways (146). Inflammatory pathway is the main pathway, one is the classical inflammatory pathway mediated by caspase-1, the other is the non-classical inflammatory pathway mediated by caspase-11. Inflammatory pathway of pyroptosis is an effective inducer of pro-inflammatory pathways in IS, occurring after the assembly and activation of inflammasomes (147). Inflammasomes contain pattern recognition receptors (PRRs), adapter proteins, and caspase family members (148). The adapter protein ASC has a cysteine protease recruitment domain (CARD) and a pyrin domain (PYD) (149). The structural characteristics of ASC provide support for binding of procaspase-1 to receptors. After IS, DAMPs secreted by necrotic cells in the ischemic core region are recognized by PRRs. Then, procaspase-1 autocrine signaling produces cleaved caspase-1. Cleaved caspase-1 mediates microglial pyroptosis with the release of a large number of pro-inflammatory factors (IL-1 and IL-18) that induces neuronal death (150). Caspase-1 cleaves gasdermin D (GSDMD) into N-GSDMD, which binds directly to the plasma membrane and forms pores, releasing large amounts of cytosolic content to promote inflammation. The non-classical inflammatory pathway is one in which caspase-11 mediates the “non-canonical inflammasome” to participate in IL-1 and IL-18 processing and cell death (108). Studies had shown that ccaspase-11 (mouse-derived) also cleaves GSDMD, leading to focal ptosis under LPS stimulation (151). In addition, caspase-11 has been found to promote inflammation by regulating caspase-1 expression by promoting K^+^ efflux (152). Non-inflammatory pyroptosis pathways are pathways in which caspase-8 is involved. It has been found that catalytic caspase-8 promotes the assembly of ASC-procaspase-1, in which caspase-8 acts as a scaffolding protein (153). In addition, caspase 8, procaspase 1, and cleaved caspase-1 were upregulated in an MCAO/R model (154). Similar to caspase-1, caspase-8 can also cleave gasdermin family proteins to induce pyroptosis. Under hypoxia conditions, nuclear transcription of GSDMC increases and caspase-8 cleaves GSDMC into N-GSDMC to induce pyroptosis after TNF-α stimulation (155). Further studies have identified a principal axis of pyroptosis extending from ROS-initiated DR6 endocytosis to caspase 8-mediated GSDMC cleavage (156).

#### 3.1.2 Apoptosis

Apoptosis is the most common form of programmed cell death in multicellular organisms that elicits no inflammatory response. It is the main mechanism of neuronal loss after IS and can be triggered either through the intrinsic or the extrinsic pathway. The intrinsic pathway is caused by DNA damage or endoplasmic reticulum stress, and the extrinsic pathway is mediated by the activation of the death receptor family members (157).

The intrinsic pathway involves a non-receptor-mediated signaling cascade (158). After IS, excitotoxicity produced through the mitochondrial pathway can mediate Ca^2+^ overload, leading to cell apoptosis. Glutamate binds to N-methyl-D-aspartate receptors (NMDARs), resulting in an overload of Ca^2+^ in neurons (159). Ca^2+^ activates the interaction of calpain with the Bcl-2 family proteins. Eventually, proapoptotic proteins are upregulated and mitochondrial permeability transition pores are formed (160), allowing for the release of apoptogens. The Bcl-2 protein family members (pro-apoptotic) regulate changes in the mitochondrial permeability, the release of cytochrome c, and contribute to apoptogen formation by binding with apoptotic protease activating factor-1 (Apaf-1) (161). Finally, activation of caspase leads to degradation of nuclear DNA, thus promoting cell apoptosis (162).

Following IS, the activation of immune cells during inflammation results in the release of a variety of factors (including pro-inflammatory cytokines) that trigger neuronal cell death via the extrinsic apoptotic pathways (163, 164). The extrinsic cell apoptosis pathway is triggered by the ligation of tumor necrosis factor (TNF)-family death receptors on the cell surface during external stimuli (165). After the receptor is bound, it recruits the adapter protein [Fas-associated death domain protein (FADD)] to create a death‐inducing signaling complex with procaspase-8, which activates caspase-8 (166). Caspase-8 activates the downstream effector caspase, mediating apoptosis by direct proteolytic cleavage or indirectly by catalyzing the Bcl-2 protein family members (167). Velier et al. established a pMCAO rat model and found that proteolytic processing yielding the active form of caspase-8 was active 6 hours after stroke (168).

#### 3.1.3 Necroptosis

Necroptosis, which is a lytic-programmed cell death with the ability to cause inflammation, is independent of caspase transmission. Similar to apoptosis, necroptosis is triggered by the ligation of specific death ligands to TNF-family death receptors or by pro‐caspase inhibitors (169). This process leads to de-ubiquitination of receptor interacting protein kinase 1 (RIPK1) by the de-ubiquitination enzyme CYLD (170). RIPK1 activates the kinase RIPK3 within a cytoplasmic high molecular weight complex called a necrosome. RIPK3 phosphorylates and activates the mixed lineage kinase domain-like protein (MLKL), forming the homotrimer necrosomes (171). The accumulation of necrosomes leads to increased permeability of plasma membranes and organelles. This leads to membrane damage and subsequent cell death (172, 173). The phosphorylation of MLKL and the formation of necrotic bodies are therefore considered as cellular markers of necrosis (174). After cerebral I/R injury, perivascular M1-microglia secrete TNF-α and its receptor TNFR1 on the endothelium, which serve as the main mediators triggering endothelial necroptosis (175). Necroptosis promotes the release of DAMPs, driving an inflammatory response. *In vivo* and *in vitro* studies found that RIPK3 promoted NLRP3 inflammasomes and the IL-1β inflammatory response independently of MLKL and necroptosis (176). In another study, it was found that MLKL signaling also activated NLRP3 inflammasomes and induced IL-1β secretion to promote inflammation. MLKL-induced NLRP3 inflammasome formation and IL-1β cleavage occur before cell lysis (177).

#### 3.1.4 Ferroptosis

Ferroptosis refers to a new form of cell death caused by an increase of iron ion-dependent lipid peroxide (178). It is characterized by the accumulation of iron-regulated lipid peroxidation and caused by an imbalance of lipid metabolism, the depletion of glutathione (GSH), and the abnormal metabolism of iron. Excessive accumulation of iron is the key feature of ferroptosis, and most iron comes from damaged or aged red blood cells. Fe^2+^ produced by erythrocyte degradation can be oxidized to Fe^3+^, and Fe^3+^ binding transferrin (TF) mediates endocytosis through transferrin receptor (TFR)1 (179, 180). After the endocytosis of TF-TFR1, Fe^3+^ is released from TF and reduced to Fe^2+^ by six-transmembrane epithelial antigen of the prostate 3 (STEAP3). Finally, unbound iron is easily absorbed by neurons, resulting in intracellular iron accumulation (181). When iron is overloaded, Fe^2+^ generates a large number of lipid-active oxygen radicals through the Fenton reaction. Fe^2+^ can also participate in the synthesis of lipoxygenase and then catalyzes lipid peroxidation (182).

Lipid peroxidation is a critical process of ferroptosis (183). Ferroptosis shows obvious lipid peroxidation stress and cell membrane damage. Polyunsaturated fatty acid (PUFA)-phospholipid (PL) species are the most sensitive to peroxide because they contain highly active hydrogen atoms in their methylene bridge. In ferroptosis, acyl-CoA synthetase long-chain family member 4 (ACSL4) catalyzes fatty acids to form acyl coenzyme A and promotes fatty acid oxidation or lipid biosynthesis (184). Next, lysophosphatidylcholine acyltransferase 3 (LPCAT3) inserts the composite into the membrane phosphatidylethanolamine (PE). The ferroptotic signal is then activated (185). These lipids can be peroxidized under the catalysis of lipoxygenase (LOX) or under the induction of ROS (OH^-^) produced in the Fenton reaction. The resulting lipid peroxide can attack the proximal PUFA, causing a chain reaction and ferroptosis (186).

The manifestations of ferroptosis is the depletion of GSH and the inactivation of glutathione peroxidase 4 (GPX4). GSH is a tripeptide containing a sulfhydryl group, and it is composed of glutamic acid, glycine, and cysteine. It can combine with free radicals to repair cell membrane damage caused by lipid peroxide and it can clear ROS (187). GPX4 is a selenium enzyme. It can reduce oxidized lipids (L-OOH) (such as cholesterol and PL containing PUFA) to harmless lipid alcohols (L-OH) by converting GSH into oxidized glutathione (GSSG) (188). Therefore, GSH can also regulate GPX4 activity. In the process of ferroptosis, the accumulation of oxidation-reduction active iron consumes GSH reserves through the Fenton reaction, and then inhibits the activity of GPX4, leading to an overwhelming antioxidant reaction (189). The lack of GPX4 in turn leads to the accumulation of iron.

#### 3.1.5 PANoptosis

Pyroptosis and apoptosis both involve the activation of members of the caspase protease family. Studies have found that the activation of caspase-1 triggers pyroptosis and apoptosis (190). As has already been mentioned, RIPK3 and MLKL are crucial for the occurrence of necroptosis. They can also mediate the formation of NLRP3 inflammasomes and trigger pyroptosis. This widespread crosstalk between pyroptosis, apoptosis, and necroptosis led to a new form of programmed cell death called “PANoptosis”. PANoptosis is an inflammation-regulated cell death pathway. These cell death pathways are interconnected through the shared regulatory proteins called the PANoptosome. The PANoptosome is a cell death–inducing complex that is characterized by pyroptosis, apoptosis, and necroptosis molecules. It was identified as an inducer and regulator of PANoptosis. Christgen et al. found that RIPK1, RIPK3, caspase-8, NLRP3, ASC, and FADD interacted to form PANoptosomes (191). These proteins can be divided into sensors (ZBP1 and NLRP3), adapters (ASC and FADD), and catalytic effectors (RIPK1, RIPK3, caspase-1, and caspase-8) based on their functions (192). Lee et al. found that AIM2 regulated the innate immune sensors pyrin and ZBP1 to drive inflammation signal transduction and PANoptosis. The results confirmed that AIM2 mediated the assembly of multi-protein complexes, known as the AIM2 PANoptosome (193). Another study found a RIPK1 PANoptosome complex in an *in vivo* model of bacterial pathogen infection, which regulates all three branches of PANoptosis (194). In addition, during influenza virus infection, ZBP1 recruited RIPK3 and caspase-8 to activate ZBP1-NLRP3 inflammasomes. The formation of ZBP1-NLRP3 inflammasomes mediates PANoptosis by assembling the ZBP1 PANoptosome (195). Yan et al. confirmed the existence of PANoptosis in *in vitro* and *in vivo* models of ischemic brain injury through researching literature (196). In a following study, they demonstrated the occurrence of PANoptosis-like cell death in *in vivo* and *in vitro* models of ischemia/reperfusion injury (197). In summary, these studies indicate the presence of PANoptosis in ischemic brain injury. However, more research is needed to broaden our understanding of the basic processes of neuronal cell death and molecular targets, and to identify key molecules that regulate PANoptosis, which will lead to the development of new therapies.

## 4 Regulation mechanisms of natural compounds

The pathology of ischemic brain injury is an exceptionally complex pathological process involving a variety of cytotoxic factors and inflammatory cells in the CNS as well as in the peripheral circulatory system. Inflammation and cell death are the two main factors in IS. Inflammation and cell death, which are caused by ischemia, overlap and are interrelated. Due to the complexity of these factors and their interactions, it is very difficult to develop effective treatment methods based on the “one drug, one target” strategy, which leads to adverse outcomes in stroke treatment (198). A substantial number of studies have shown that some natural compounds (such as salidroside, baicalin, astragaloside IV, and curcumin) have protective effects on IS with few side effects (shown in **Table 4**).

**Table 4:** Regulation mechanisms of natural compounds.

Components	Experimental model	Effective dosage	Treatment time point and path	Targets	Results
Salidroside	Rats (MCAO), SH-SY5Y (I/R)	20 or 40 mg/kg	Before 30 minutes surgery (administrate orally)	↓ TNF-α, IL-1β, IL-6 and Bcl-2 ↑ RIP140, Bax, p-IKKα, p-IKKβ, p-IκBα, and p-p65	Anti-inflammation, inhibit RIP140/NF-κB pathway, anti-apoptosis,
	BV2 microglial cells (LPS)	75, 150, and 300 μM	After LPS	↓ MCP-1, MIP-1α, and IL-8 ↓ d-p-IκBα, p-NF-κB ↓ p65, p-JNK, p-p38 and p-ERK1/2	Anti-inflammation, inhibit migratory ability of BV2 cells,
	Rats (2/1h MCAO/R)	50 mg/kg	After MCAO/R (i.p.)	↓ TNF-α, IL-1β, IL-6, CD14, CD44, iNOs, CD11b, ↑ NeuN, p-Akt, HIF1α, HIF2α, HIF3α, EPO	Anti-inflammation, inhibit PI3K/Akt signaling
	Rats (pMCAO)	100 mg/kg	7 days i.p.	↑ NeuN, Nrf2, HO-1, p-PKB ↓ NF-κB p50, IL-6, TNF-α	Anti-inflammation, activate PI3K/PKB signaling pathway
	Mice (tMCAO/R 1h)	2.5, 5, 10, and 20 mg/kg/day	Give immediately after R (CVI) once/day for 5 days	↓ TNF-α, IL-1β, IL-2, IL-6, and IL-8 ↑ CD206, Arg1, TGF-β, and YM1/2	Anti-inflammation, promote M2 microglial polarization inhibit M1 microglial polarization
	Rats (MCAO/R) HUVEC (OGD/OGD-OGR)	50 mg/kg (rat), 10μM (cell)	After MCAO and OGD/OGD-OGR	↑ CD46, CD59 ↓ ICAM-1, VCAM-1, P-selectin, and E-selectin ↓ C1q, C2-mRNA, C3 protein level ↓ Bcl-2/Bax	Anti-inflammation, anti-apoptosis, suppress endothelial activation, inhibit neutrophilic recruitment
	MCAO mice (2/24h MCAO/R) neurons cells (OGD/R)	25, 50, and 100mg/kg	3 days/once (i.p.)	↑BDNF, p-PI3K, p-AKT ↑p-Bad, Bcl-2 and Bcl-xl	Anti-apoptosis, inhibit BDNF/TrkB/AKT/FoxO1 pathway, decrease accumulation of FoxO1,
	PC12 cells (H_2_O_2_ 12h)	100µM	Pretreat	↑ Bcl-2 ↓ Bax ↓ cytochrome C release ↓ caspase-3, caspase-8 and caspase-9	Anti-apoptosis
	NGF-differentiated PC12 cells (H_2_O_2_ 90 min)	128 µM	Pretreat 24h	↓ caspase-3, p-ERK1/2	Anti-apoptosis, activate ERK pathway
	Rats (2/24h MACO/R)	12mg/kg	Pretreat 7 days/once	↓ ROS, Bax ↑ Bcl-2	Anti-apoptosis, anti-oxidative effect,
Astragaloside IV	Rats (MCAO/R, after 1h MCAO to achieve R)	12.5 mg/kg, 25 mg/kg, and 50mg/kg	7 days/once after surgey (i.g.)	↓Fas, FasL, and Bax/Bcl-2 ↓caspase-8, Bid, cleaved caspase-3 and cytochrome C	Anti-apoptosis, inhibit death receptor pathway
	Rat primary cultured astrocyte (OGD/R)	16 μM, 32 μM, and 64 μM	After 4 h OGD	↓Bax/Bcl-2, Keap 1, ↑Nrf2, p-JNK/JNK	Anti-apoptosis, anti-oxidative effect, inhibit CXCR4/JNK pathway, and upregulate Keap1/Nrf2 pathway
	Mice (PBI), NSCs	200 mg/kg	3 days/once after stroke (i.v.)	↓IL-17, caspase 3, and number of NeuN/TUNEL ↑p-PI3K, p-Akt, and numbers of DCX/BrdU ↑numbers of Wnt2^+^ cells	Anti-apoptosis, upregulate Akt/GSK-3β pathway, upregulate Wnt/β-catenin pathway
	Mice (photothrombosis), NSCs	2 mg/kg	3 days/once after stroke (i.v.)	↑DCX/BrdU and Sox2/Nestin ↓IL-17 ↑Wnt2, β-catenin, and GSK-3β,	Promote neurogenesis, activate NSC proliferation, upregulate Wnt pathway
	Rats (MCAO/R) PC12 cells (OGD/R)	20 mg/(kg)	During reperfusion (i.p.)	↓cleaved caspase-3, AIF, and CaSR ↓Bax/Bcl-2	Anti-apoptosis, decrease the apoptotic rate, and inhibit calcium overload,
	Rats (MCAO) HUVECs (OGD/R)	40 mg/kg	Immediately after MCAO (i.g.)	↓EphrinA3 ↑miRNA-210	Activate HIF/VEGF/Notch pathway, stimulate angiogenesis
	Rats (tMCAO)	40mg/kg	14 days/once after MCAO (i.p.)	↑PPARγ, BDNF, IGF-1, and VEGF ↓CD86, iNOS, TNF-α, IL-1β, IL-6 ↑CD206, Arg-1, YM1/2, IL-10, TGF-β	Anti-inflammatory, promote M1 microglia to M2 through PPARγ pathway, promote neurogenesis and angiogenesis through PPARγ pathway
	Rats (MCAO)	40 mg/4 ml/kg	14 days/once (i.p.)	↑BDNF, TrkB	Promote neurogenesis, upregulate BDNF/TrkB pathway,
	Mice (MCAO) Primary cortical neurons (OGD/R)	15 and 30 mg/kg	/	↓cytochrome C, TUNEL-positive cells, glutamate, and caspase-3 ↑NAD^+^ and ATP,	Anti-apoptosis, promote HK-II binding to mitochondria through Akt, protect mitochondrial integrity
	Primary cerebral cortical neurons (OGD/R)	6.25, 12.5 and 25 μmol/L	At the start of OGD/R	↓Cleaved caspase-3, ↑ATP, p-CREB, PKA	Anti-apoptosis, activate PKA/CREB pathway, protect mitochondrial,
Baicalin	Rats (pMCAO 24h)	30 or 100 mg/kg	2 and 12 h twice after the onset of ischemia (i.p.)	↓COX-2, iNOS, MPO, cleaved caspase-3, TUNEL-positive cells	Anti-apoptosis, reduced cerebral infarct area and infarct volume
	Rats (pMCAO 24h)	100 mg/kg	2 and 12 h twice after the onset of ischemia (i.p.)	↓TLR2/4, NF-κB, TNF-α, IL-1β ↓NF-κB p65, iNOS, COX-2	Anti-inflammation, reduced cerebral infarct area and infarct volume, inhibit activity of iNOS, COX-2
	Rats (pMCAO 24h)	100 mg/kg	2 and 12 h twice after the onset of ischemia (i.p.)	↓MMP-9 ↓expression of occludin	Anti-inflammation, reduced brain edema and BBB permeability
	Rats (MCAO/R), 2/24h, P12 cells (OGD/R)	100 mg/kg	24h	↓Drp-1, ↑ MFN2	Anti-apoptosis, enhanced mitophagy,
	Seven-day-old baby rats (left common carotid artery ligation)	120 mg/kg	After hypoxia for 2 h(i.p.)	↑p-Akt, GLT-1	Anti-apoptosis, upregulate GLT-1 via the PI3K/Akt pathway
	Rats (MCAO/R), primary astrocytes (OGD/R)	50 mg/kg	30 min before R (i.p.)	↓Mitochondrial succinate dehydrogenase	Anti-apoptosis
Curcumin	Rats (MCAO/R) 2/22h	200 mg/kg	30 min after I/R (i.p.)	↑p-Akt, p-mTOR, ↓LC3-II/LC3-I, IL-1, TLR4, p-38, and p-p38 ↓IL-6, TNF-α, and iNOS	Anti-inflammation, regulate TLR4/p38/MAPK pathway, mediate PI3K/Akt/mTOR pathway, improve neurological functions and reduce cerebral infarction,
	Rats (MCAO/R)	25 mg/kg	After MCAO (i.p.)	↑Bcl-2, Sirt1 ↓MMP, p53 and Bax ↓IL-6, TNF-α	Anti-inflammation, anti-apoptosis, reduce mitochondrial dysfunction, reduce infarct volumes and brain edema
	Mice (dMCAO/R) BV2 microglia (LPS, IFN-γ)	150mg/kg	0 h and 24 h after reperfusion (i.p.)	↓IL-6, TNF-α, IL-12p70 ↓CD16, CD32, iNOS ↑Arg-1 and YM1/2	Anti-inflammation, inhibit M1 microglia polarization, promote M2 microglia polarization
	Rats (MCAO)	300 mg/kg	7 days/once after stroke (i.p.)	↑BrdU-positive cells, ↑BrdU/DCX-positive cells ↑NICD	Activate Notch pathway, improve neurofunctional recovery, promote neurogenesis
	Rats (MCAO)	300 mg/kg	30 min after MCAO (i.p.)	↓NF-κB, ICAM-1, MMP-9, ↓caspase-3	Anti-inflammation
	Mice (MCAO/R) N2a cells (OGD/R)	100, 200, 300 and 400 mg/kg	After occlusion 1 h (i.p.)	↓Bax, cleaved caspase-3 ↑Bcl-2,	Anti-apoptosis, alleviate mitochondrial dysfunction,
	Rats (MCAO/R)	50 mg/kg	5 days/once before MCAO (i.p.)	↑Sirt1, Bcl-2 ↓Ac-p53, Bax, cytochrome c ↓IL-6, TNF-α	Anti-apoptosis, anti-inflammation
	Primary cortical neurons (OGD/R)	0.25-10 μM	Add to culture medium	↓LDH, caspase-3, p-JNK ↑flotillin-1, p-ERK1/2	Attenuate cell death, regulate flotillin-1 and MAPK/ERK pathway,

References are shown in text.

nuclear receptor-interacting protein 1 (RIP40), phosphorylation (p-), lipopolysaccharide (LPS), degradation and phosphorylation (d-p-), phosphorylated protein kinase B (p-Akt), hypoxia-inducible factor (HIF) subunits (HIF1α, HIF2α, HIF3α), erythropoietin (EPO), intraperitoneal (i.p.), reperfusion (R), caudal vein injection (CVI), oxygen-glucose deprivation followed by restoration (OGD-OGR), superoxide dismutase (SOD), glutathione-S-transferase (GST), malondialdehyde (MDA), human umbilical vein endothelial cell (HUVEC), ultraviolet B (UVB), sunburn cells (SBCs), 8-hydroxy-2’-deoxyguanosine (8-OHdG), transient MCAO (tMACO), Kelch-like ECH-associated protein-1 (Keap 1), oxygen glucose deprivation/reoxygenation (OGD/R), C-X-C motif chemokine receptor 4 (CXCR4), photochemical brain ischemia (PBI), neural stem cells (NSCs), injected intravenously (iv.), phosphatidylinositol-4,5-bisphosphate 3-kinase (PI3K), glycogen synthase kinase-3β (GSK-3β), doublecortin (DCX), S-phase marker 5-bromo-2′-deoxyuridine (BrdU), pheochromocytoma (PC12), calcium−sensing receptor (CaSR), apoptosis-inducing factor (AIF), peroxisome proliferator-activated receptor γ (PPARγ), vascular endothelial growth factor (VEGF), brain-derived growth factor (BDNF), insulin-like growth factor-1 (IGF-1), vascular endothelial growth factor (VEGF), wingless/integrated (Wnt), hexokinase II (HK-II), protein kinase A (PKA), cyclic AMP response element-binding protein (CREB), permanent middle cerebral artery occlusion (pMCAO), intraperitoneally injected (i.p.), myeloperoxidase (MPO), inducible nitric oxide synthase (iNOS), cyclooxygenase-2 (COX-2), toll-like receptor 2 and 4 (TLR2/4), nuclear factor-kappa B (NF-κB), tumor necrosis factor-alpha (TNF-α), interleukin-1β (IL-1β), streptozotocin (STZ), oxygen-glucose deprivation/reperfusion (OGD/R), mitofusin-2 (MFN2), glutamate transporter 1 (GLT-1), phosphoinositide 3-kinase/protein kinase B (PI3K/Akt), NAD-dependent protein deacetylase sirtuin-1 (Sirt1), distal middle cerebral artery occlusion (dMCAO), arginine-glutamic acid dipeptide repeats protein (Arg-1), Notch intracellular domain (NICD), silent information regulator 1 (Sirt1), acetylated p53 (Ac-p53). The meaning of the symbol "↑" is “upregulated”. The meaning of the symbol “↓” is “downregulated”. /, not appicable.

### 4.1 Salidroside

Salidroside (Sal) is the main bioactive component in Rhodiola rosea. In many studies of IS *in vitro* and *in vivo* in cells and animals, salidroside has demonstrated strong biological activity. Sal can significantly reduce the brain infarct size and cerebral edema by inhibiting inflammatory signaling. Sal reduces the levels of pro-inflammatory cytokines and chemokines in tissues or serum, such as TNF-α, IL-2, IL-6, IL-8, IL-1β, MCP-1, and MIP-1α (199). After cerebral ischemia, inflammatory transduction mainly depends on NF-κB, mitogen-activated protein kinases (MAPK), phosphatidylinositol 3 kinase/protein kinase B (PI3K/Akt), and phosphoinositide 3-kinase/protein kinase B (PI3K/PKB) signaling pathways. Chen et al. found Sal effectively reduced the levels of IL-6, IL-1β, and TNF-α by blocking the RIP140/NF-κB pathway (200). Sal also significantly inhibited activation of NF-κB, blocked degradation of tropomyosin-related kinase B (IκBα), and reduced p-MAPK levels (JNK, p38 and ERK1/2) (201). In a further study, it was demonstrated that Sal inhibited CD11b and inflammatory mediators through PI3K/Akt/HIF signaling. Sal significantly upregulated HIF subunits (HIF1α, HIF2α, and HIF3α) and the HIF downstream target (erythropoietin). Sal reduced CD14, CD44, and iNOS mRNA (202). Zhang et al. demonstrated that Sal reduced inflammation and brain damage through PI3K/PKB/Nrf-2/NFκB signaling transduction. Sal induced NeuN and inhibited NF-κB p50 subunit and other pro-inflammatory mediators. It prevented a significant decrease in the proportion of p-PKB/PKB in the brain (203). These studies imply that Sal may inhibit inflammatory signaling through the Nrf2, HIF, MAPK, PI3K/Akt, PI3K/PKB, and NF-κB signaling pathways. In addition, Sal acts on immune cells to recover the damage caused by IS. A recent study reported that Sal significantly inhibited the release of inflammatory factors derived from microglia. To study microglia polarization, M1 phenotypic markers (CD16, CD32, iNOS, and CD11b) and M2 phenotypic markers (CD206, Arg1, TGF-β, and YM1/2) were analyzed. The results showed that Sal promoted the transformation of microglia from the M1 phenotype to the M2 phenotype to enhance the phagocytosis of microglia. At the same time, Sal-treated M1 microglia promoted oligodendrocyte differentiation (204). Sal has been shown to effectively reduce VCAM-1, ICAM-1, P-selectin, and E-selectin, as well as neutrophil recruitment in the ischemic brain (205).

Apoptosis is one of the main mechanisms of brain injury, and Sal has been found to have significant anti apoptotic effects. Brain-derived neurotrophic factor (BDNF) is a member of the neurotrophic factors. BDNF has a protective effect on ischemic brain injury. Zhang et al. indicated that Sal produced its anti-apoptotic effects by regulating the BDNF-mediated PI3K/Akt apoptosis pathway in a DNA-binding-dependent and -independent manners (206). Sal has been shown to inhibit the downregulation of Bcl-2, the upregulation of Bax, and the release of mitochondrial cytochrome c into the cytosol. Sal attenuated the activation of caspase-3, -8, and -9, and ultimately protected cells from apoptosis (207). Another study demonstrated that Sal induced activation of the mitogen-activated protein kinase kinase (MAPKK)/extracellular signal-related protein kinase (ERK) pathway, thereby reducing cell apoptosis (208). Shi et al. showed that Sal decreased the expression of Bax and restored the balance between pro-apoptotic and anti-apoptotic proteins (209).

**Figure 3:**
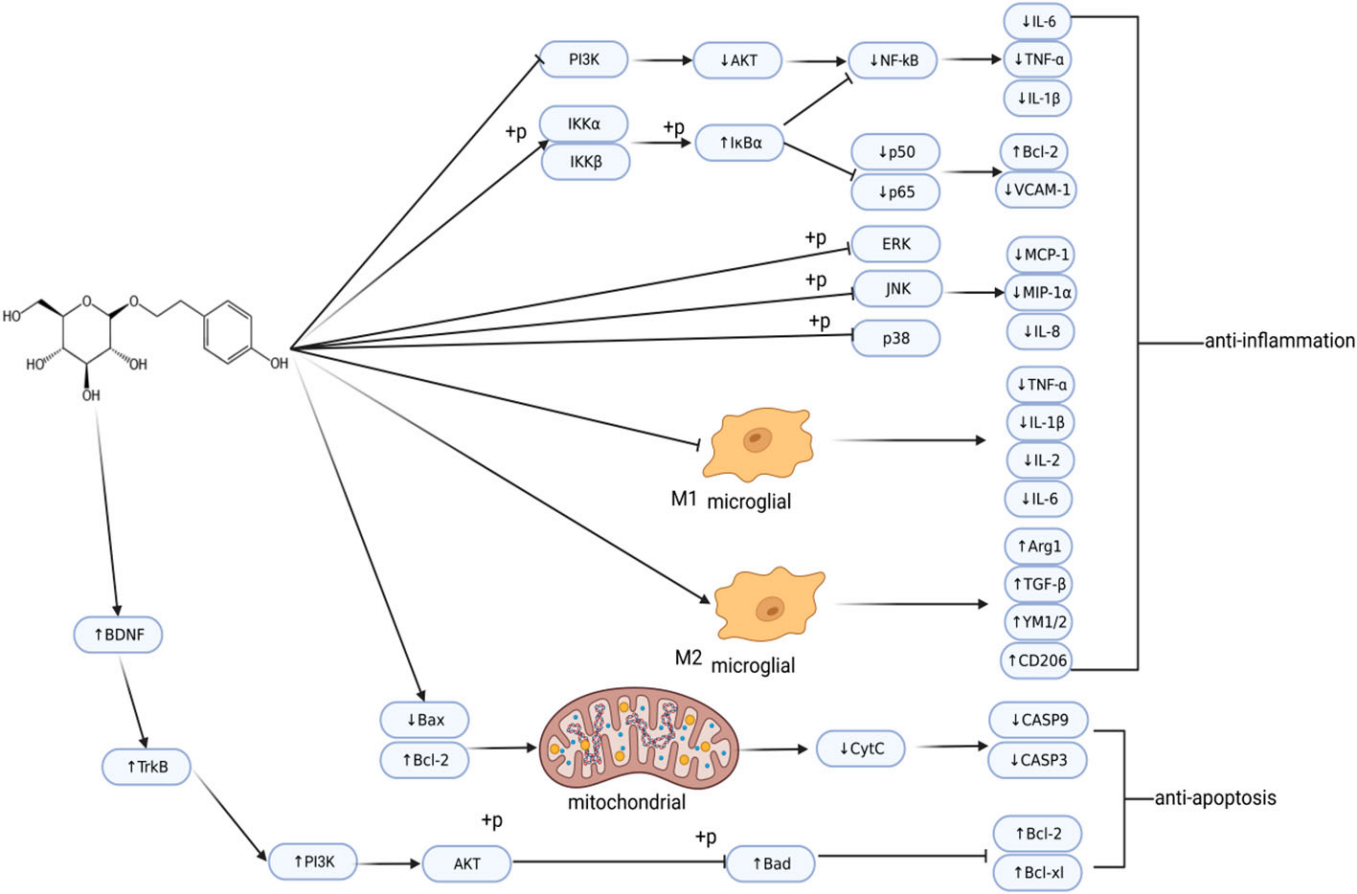
Regulatory network of salidroside in ischemic stroke. Post-IS, salidroside regulates inflammation and cell apoptosis. This image was created using BioRender.com.

These studies indicate that Sal has anti-inflammatory and anti-apoptotic effects (**Figure 3**). In addition, Sal also demonstrated excitotoxicity inhibition and anti-oxidant effects, and reduced damage to the BBB. Therefore, as an effective neuroprotective agent, it can be developed as a potential drug for treating stroke.

### 4.2 Astragaloside IV

Astragaloside IV (AS-IV) is one of the main active ingredients from Astragalus (Astragalus membranaceus (Fisch.) Bunge., Leguminosae, Huangqi in Chinese). AS-IV has been shown to significantly reduce neuronal apoptosis. AS-IV can suppress the activation of key factors in the death receptor pathway. AS-IV was found to inhibit mRNA upregulation of Fas, FasL, Caspase-8, and Bax/Bcl-2. AS-IV also inhibited the protein levels of caspase-8, Bid, cleaved caspase-3 and cytochrome C (210). AS-IV can regulate the Nrf2 signaling pathway. Yang et al. found that AS-IV induced Nrf2 through the downstream signaling pathways (MAPK pathway) to prevent cell apoptosis. AS-IV inhibited the CXCR4 receptor and downregulated the activation of the p-JNK/JNK pathway, thereby inhibiting the expression of Bax/Bcl-2 and ultimately increasing Nrf2/Keap1 signaling (211). Another study confirmed that AS-IV regulated cell apoptosis through the PI3K/Akt/GSK-3β pathway (212). The calcium-sensing receptor (CaSR) is a G-protein-coupled receptor. Its activation can increase the intracellular calcium concentration and contribute to cell apoptosis (213). AS-IV alleviated brain injury by inhibiting cell apoptosis induced by CaSR activation (214). Excitotoxicity by glutamate and mitochondrial dysfunction are common causes of cell apoptosis. AS-IV protects the integrity of mitochondria by promoting the combination of Akt and hexokinase II (HK-II). This helps to protect neurons from cell apoptosis and DNA damage (215). The PKA/CREB pathway regulates mitochondrial activity. AS-IV protects primary neurons from IS-induced apoptosis by regulating the PKA/CREB pathway and protecting mitochondrial function (216).

AS-IV also promotes the conversion of immune cells to an anti-inflammatory phenotype after IS, thereby reducing brain damage. PPARγ is a nuclear transcriptional factor that is widely expressed in microglia (217). AS-IV can promote the polarization of M1 microglia to the M2 phenotype, which plays a long-term neuroprotective role in cerebral ischemia/reperfusion injury through the PPARγ pathway (218). In addition, AS-IV also promotes angiogenesis by activating the HIF/VEGF/Notch, Wnt, and BDNF-TrkB pathways after IS, increasing cell proliferation, migration, and neovascularization (219–221).

### 4.3 Baicalin

Baicalin (BA) is a natural flavonoid compound isolated from the dried roots of Scutellaria baicalensis Georgi. BA alleviates the inflammatory reaction. BA was found to inhibit the TLR2/4 signaling pathway during cerebral ischemia, reducing expression of TLR2/4 and NF-κB in rat brain tissue. BA also attenuated the serum levels of TNF-α and IL-1β (222). In subsequent studies, Tu et al. also found that administration of BA after focal cerebral ischemia significantly reduced brain edema and BBB permeability. Overexpression of MMP-9 degraded the tight junction protein occludin, disrupting the integrity of the tight junction of the BBB (223). BA significantly downregulates the expression of MMP-9 protein and mRNA (224).

A growing body of evidence has shown the beneficial roles of BA in stroke management, such as anti-apoptosis. BA significantly inhibited neuronal apoptosis after cerebral ischemia injury in rats. Tu et al. found that BA significantly decreased MPO enzyme activity and iNOS and COX-2 mRNA expression in rat brain tissue, and significantly inhibited the expression of cleaved caspase-3 protein after IS (225). Li et al. showed that BA inhibited the expression of dynein related protein 1 (Drp-1). BA also reduced mitochondrial division and promoted the production of mitochondrial fusion protein 2 (MFN2) in an AMPK-dependent manner (226). Zhou et al. found that BA activated Akt phosphorylation and upregulated glutamate transporter 1 (GLT-1) expression through the PI3K/Akt signaling pathway. This inhibited cell apoptosis and reduced cerebral infarction volume and neuronal loss (227). BA also reduced mitochondrial succinate dehydrogenase (SDH)-mediated oxidative stress and reduced subsequent loss of glutamine synthetase (GS) (228).

### 4.4 Curcumin

Curcumin (CCM) is a compound mainly extracted from Curcuma longa. After IS, CCM can attenuate the inflammatory effect. The MAPK signaling pathway is regulated by TLR4 signaling and plays a key inflammatory role in IS. CCM alleviates inflammation of IS through the TLR4/p38/MAPK pathway. After CCM treatment, the protein levels of TLR4, p-p38, and IL-1 decreased, while the expression of IL-6, TNF-α, and iNOS increased (229). Another study demonstrated that CCM reduced inflammation by reducing levels of pro-inflammatory cytokines. Simultaneously, mitochondrial function was restored through an increase of MMP (230). In addition, CCM has a profound regulatory effect on the microglial response, promoting M2 microglia polarization and inhibiting the microglia-mediated proinflammatory response (231). However, further research is needed to confirm the involvement of curcumin and the specific mechanism of microglia phenotype regulation using stroke models. At the transcriptional level, the activation of NF-κB regulates ICAM-1, MMP-9 and caspase-3 expression (232). CCM decreased the expression of NF-κB, and subsequently attenuated the expression of the downstream mediators ICAM-1, MMP-9, and caspase-3 (233).

CCM exerts neuroprotective effects on IS and inhibits cell apoptosis. CCM reduces mitochondrial dysfunction and inhibits apoptosis by maintaining mitochondrial membrane potential and inhibiting the upregulation expression of Bax and downregulation of Bcl-2 (234). Silent information regulator 1 (Sirt1) is a class III group histone deacetylases that can protect the brain from ischemic damage (235). CCM was found to upregulate the expression of Sirt1 and Bcl-2 and downregulate the expression of acetylated p53 (Ac-p53) and Bax. Activating Sirt1 weakened cell apoptosis and promoted the neuroprotective effect of CCM (236). The MAPK signaling pathway regulates the expression of various pro-inflammatory cytokines and mediates apoptosis after ischemic injury. ERK1/2 and JNK are two of the main effectors of the MAPK signaling pathways (237). Lu et al. found that CCM reduced p-ERK1/2 and increased p-JNK protein levels. CCM also increased the level of flotilin-1 protein, thereby reducing cell death (238). CCM also improved neurofunctional recovery and promoted neurogenesis through Notch signaling after IS (239).

Therefore, based on the anti-inflammatory and anti-apoptosis effects of CCM, it may be a useful and promising neuroprotective agent against acute IS.

## 5 Conclusions and perspectives

Immunity, inflammation, and cell death play critical roles in the occurrence and development of stroke. This review summarizes the roles and mechanisms of immune cells and cell death pathways in IS. The immune cells discussed included microglia, astrocyte, neutrophils, T lymphocytes, and monocytes/macrophages. The cell death pathways discussed included apoptosis, pyroptosis, necroptosis, PANoptosis, and ferroptosis. This review also summarized the mechanisms of natural compounds in the treatment of IS. The natural compounds discussed include salidroside, baicalin, astragaloside IV, and curcumin.

Microglia and monocytes/macrophages form the first line of defense, but are involved in damage in the early stages of ischemic stroke. Microglia can induce increased damage to the A1 neurotoxic subtype of astrocytes. Neutrophils are recruited into damaged brain tissue, which can exacerbate inflammation. Subsequently, microglia and monocytes/macrophages show anti-inflammatory and repair functions. After T cells migrate to the brain parenchyma, they differentiate into different functional types. Hence, time-defined treatments targeting different phenotypes of immune cells may provide a clear protective strategy. At the same time, the interactions between immune cells cannot be ignored. The mutual coordination between immune cells is also caused by various inflammatory mediators. After IS, peripheral immune cells and brain immune cells form a complex inflammatory network. Treatments that target only one type of immune cell may be harmful or offset the benefits of another type of immune cell, resulting in an unsatisfactory stroke prognosis. Therefore, therapeutic strategies to modulate the immune system need to be further explored to determine effective treatment measures.

Compared to PANoptosis, the key molecular pathways involved in apoptosis, pyrotosis, ferroptosis, and necroptosis are clearer. Further research is needed on the molecular basis and key pathways of PANopotosis after IS. Further research on the molecular and regulatory mechanisms of PANopotosis will have new impacts on the treatment of IS. For natural compounds in this review, mechanism research of Sal is the most extensive. All the natural compounds included in this review have therapeutic effects on inhibiting inflammation and cell apoptosis in IS. Salidroside, baicalin, astragaloside IV, and curcumin may be effective and promising candidates for the treatment of IS. However, they still have certain limitations, including whether they can show the same effect clinically as in research studies. Future research directions include 1) mechanisms for drugs to enter the central nervous system, 2) the ability to penetrate the blood-brain barrier and distribute widely in the brain, and 3) the side effects of drugs. With further research, the discovery of new drugs will lead to better treatment of IS for the benefit of public health.

## Author contributions

NS: Conceptualization, Funding acquisition, Writing – review & editing. QC: Conceptualization, Writing – review & editing. ZG: Writing – original draft. JG: Writing – original draft. BL: Writing – review & editing. YG: Writing – review & editing. CC: Writing – review & editing. YJ: Writing – review & editing. NL: Writing – review & editing. MH: Investigation. TS: Writing – review & editing. LY: Writing – review & editing. HL: Writing – review & editing. HZ: Writing – review & editing. XZ: Writing – review & editing.

## Glossary

**Table:**

IS	ischemic stroke
tPA	tissue plasminogen activator
PCD	programmed cell death
DAMPs	damage-associated molecular patterns
TGF	transforming growth factor
BBB	blood brain barrier
CD11b	integrin alpha-M
CD206	macrophage mannose receptor 1
Nrf2	nuclear factor erythroid 2-related factor 2
TLR	toll-like receptor
S1PR	sphingosine 1-phosphate receptor
GFAP	glial fibrillary acidic protein
C1q	complement component subunit 1q
pMCAO	permanent middle cerebral artery occlusion
NETs	neutrophil extracellular traps
tMCAO	transient middle cerebral artery occlusion
CCR5	C-C chemokine receptor 5
CXCR1	C-X-C chemokine receptor 1
TCRs	T cell receptors
I/R	ischemia-reperfusion
CTL	CD8^+^ cytotoxic T lymphocytes
Th	CD4^+^ T helper
Treg	regulatory T
γδ	gamma delta
JAK/STAT	Janus kinase/signal transducer activator of transcription
LPS	lipopolysaccharide
IL-15	interleukin-15
IL-17	interleukin-17
PRRs	pattern recognition receptors
CARD	cysteine protease recruitment domain
PYD	pyrin domain
NMDARs	N-methyl-D-aspartate receptors
Apaf-1	apoptotic protease activating factor-1
TNF	tumor necrosis factor
FADD	Fas-associated death domain protein
RIPK1	receptor interacting protein kinase 1
MLKL	mixed lineage kinase domain-like protein
Sal	salidroside
MAPK	mitogen-activated protein kinases
PI3K/Akt	phosphatidylinositol 3 kinase/protein kinase B
PI3K/PKB	phosphoinositide 3-kinase/protein kinase B
IκBα	tropomyosin-related kinase B
BDNF	brain-derived neurotrophic factor
MAPKK	mitogen-activated protein kinase kinase
ERK	extracellular signal-related protein kinase
AS-IV	astragaloside IV
CaSR	calcium-sensing receptor
HK-II	hexokinase II
BA	baicalin
Drp-1	dynein-related protein 1
MFN2	mitochondrial fusion protein 2
GLT-1	glutamate transporter 1
SDH	succinate dehydrogenase
GS	glutamine synthetase
CCM	curcumin
Sirt1	silent information regulator 1
